# Light‐Driven Diselenide Metathesis in Peptides

**DOI:** 10.1002/open.201900224

**Published:** 2019-09-10

**Authors:** Mateusz Waliczek, Özge Pehlivan, Piotr Stefanowicz

**Affiliations:** ^1^ Faculty of Chemistry University of Wrocław Joliot-Curie 14 50-383 Wrocław Poland

**Keywords:** light-driven metathesis, peptides, mass spectrometry, diselenides, protein folding

## Abstract

Peptides containing selenocysteine moieties are susceptible to non‐catalytic reactions of diselenide bonds metathesis induced by visible light. In contrast to previously reported radical metathesis of disulfide bridges in cysteine derivatives, this newly developed reaction is fast and clean, and proceeds without decomposition of peptides and without formation of side products. The diselenide bond in peptides was reported in literature to be more stable than the disulfide one and also less susceptible to metathesis induced by thiols and reducing reagents. We demonstrated that visible light induces fast metathesis of Se−Se bonds in peptides. This reaction is important for the folding of peptides containing selenocysteine residues and may find application in designing dynamic combinatorial libraries of peptides responsive to external influence.

In the past, selenium was considered as a solely toxic element.^1^ The breakthrough occurred in 1957, when Schwartz and Foltz identified this element as an essential for bacteria, birds, and mammals.^2^ Currently, several examples of selenoproteins, such as glutathione peroxidase, have been known.^3^ Selenium occurs in proteins predominantly in a form of selenocysteine (Sec). This amino acid, found in active sites of enzymes, often acts as a nucleophile, metal ligand, or redox element.^4^ Moreover, this element attracts attention due to the similarities between cysteine and selenocysteine, which have been considered as isosteric groups.^5^ This property opened the way for more convenient chemical syntheses and was successfully utilized in synthesis of bioactive peptides, e. g. somatostatin or oxytocin.^6^ The spectroscopic analysis confirmed retention of the correct folding, bioactivity as well as the isosteric character of a diselenide bond. Armishaw *et al*. reported a beneficial impact of the Sec residue on the correct folding of α‐conotoxin.^7^ The same approach was efficient in a synthesis of biologically active and stable insulin analogue, in which one of disulfide bond connecting A and B chain was replaced by Se−Se bond.^8^ On the other hand incorporation of non‐native diselenides into proteins in some cases leads to direct folding toward formation a native‐like structures.^9^ Moreover, several low‐molecular‐weight diselenides were described as catalysts of the oxidative folding.^10^ Despite the similarity between these two amino acids, there are significant differences in their chemical properties. Sec exhibits a better nucleophilic property and constitutes, in comparison to its Cys analogue, a better leaving group. Furthermore, the Sec has been characterized as more acidic (pK_a_=5.24–5.63) than Cys, therefore, at the physiological pH undergoes deprotonation, whereas Cys remains mostly protonated.^11^ Dynamic chemistry of disulfide bond plays a crucial role when it comes to cysteine‐containing proteins and, therefore, has become a powerful tool in the dynamic covalent/combinatorial chemistry (DCC).^12^ This chemistry is used in new methods of drugs development. Among the wide scope of methods, metathesis reactions have become important tools enabling formation of e. g. two molecules from the other two.^13^ UV irradiation induced disulfide metathesis is based on homolytic cleavage of the disulfide bond and the subsequent cross‐reaction between resulting radicals, however, the visible light turned out to be sufficient for a diselenide exchange because of relatively low energy of Se−Se bond.^14^ The metathesis reaction in diselenide based systems is useful in designing self‐healing polymers^15^, ^16^ as well as in selective surface modification e. g. for bioconjugation purposes.^17^ Urs et al. found that metathesis of disulfides can be also induced by ultrasounds in chloroform solution.^18^ The reaction worked for simple low‐molecular‐weight organic compounds but the attempt at diselenide metathesis of the Boc‐protected cystine substrate was unsuccessful. Thus, according to the authors, this method is not suitable for metathesis in peptides. Rasmussen et al. described the diselenide exchange as a reversible reaction in water which works at physiological pH in a presence of thioles.^19^ Here, we present application of the light‐driven diselenide exchange to a peptide containing the selenocysteine residue (Figure 1). Additionally, we show the feasibility of the exchange reaction between a peptide diselenide and a low molecular diselenide.

**Figure 1:**
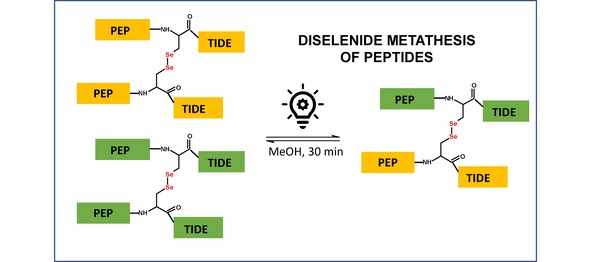
The scheme representing the exchange reaction between diselenides.

We synthesized model peptides containing the selenocysteine residue on the solid support according to a standard Fmoc protocol.^20^ For this purpose, we used the commercially available Fmoc−Sec(pMeBzl) derivative. The standard cleavage leads to a peptide with the protected Sec side chain. Therefore, the additional cleavage with 5 % DMSO/TFA for 5 h was required. This procedure results in formation of oxidized products with diselenide bridge. Purified peptides were analyzed by HPLC and the high‐resolution mass spectrometry (see Figures S1–S8). The identity of obtained compounds was manifested by the characteristic isotopic distribution, identical with the theoretical one of peptides containing two moieties of Sec. To study the metathesis reaction, we dissolved two peptides: AA (H−Ala−Ala−Sec−Lys−Lys−OH)_2_ and BB (H−Gln−Asn−Sec−Ser−Arg−OH)_2_ in methanol at the ratio of 1 : 1 (the initial concentration of both peptides was 1 mM). The sample was subsequently subjected to irradiation with a LED lamp (400–700 nm wavelength range, 1 W, 20 lumens – the spectroscopic characteristic of the lamp presented in Figure S15) for 30 min. Afterwards, the sample was analyzed by LC‐MS. We observed three abundant signals (Figure 2), which correspond to the following diselenides: AA (4.4 min), BB (5.6 min), and AB (5.0 min). The last one represents the mixed diselenide. To our surprise, the reaction proceeds with a high purity and we did not observe any by‐products which were reported during attempts at the disulfide metathesis of Boc protected cysteine.^18^ It is worth to notice that this reaction does not require a catalyst and the equilibration time is relatively short. A careful analysis of the AB peak (retention time 5 min) revealed the presence of additional signals of low abundance, corresponding to the m/z ratio of peptides AA and BB. This unexpected phenomenon can be, in our opinion, explained by the diselenide exchange which occurred in the gas phase within a high pressure region of the ESI ion source. For other investigated systems, the same process of back exchange was also observed. To check whether the diselenide metathesis of peptides will be useful for formation of peptide libraries, we carried out the experiment mixing the equimolar quantities of the following peptides: AA (H−Ala−Ala−Sec−Lys−Lys−OH)_2_, BB (H−Gln−Asn−Sec−Ser−Arg−OH)_2_, and CC (H−Ile−Leu−Lys−Glu−Pro−Val−His−Gly−Ala−Sec−NH_2_)_2_. The experimental conditions for the metathesis reaction were the same as those described for the previous experiment. The third model peptide, containing the C‐terminal Sec residue, was synthesized using a Chemmatrix® Rink amide resin. The results of acquired LC‐MS data are shown in Figure 3. According to our expectations, six peptides were observed at these conditions (signal presented as an extracted ion chromatogram XIC). An especially abundant signal, corresponding to peptides BC and AC, may indicate that the Sec residue position and the resulting sterical availability of the diselenide bridges can affect the yield of metathesis. In view of different ionization efficiency of investigated peptides, a quantitative comparison of particular diselenides in the mixture should be considered only as an approximation. However, the HPLC analysis of that sample with a UV detection at 210 nm showed a very similar composition of the sample (13.89 % AA; 7.64 % AB, 17.37 % BB; 19.23 % AC, 21.80 % BC and 20.07 % CC) to that observed by LC‐MS technique (Figure 4). We studied also the possibility of diselenides exchange between a peptide and a low‐molecular‐weight compound (1,2‐bis(4‐bromobenzyl)diselenide, see Figures S9–S11). The latter was synthesized following the procedure described in the Supplementary section.

**Figure 2:**
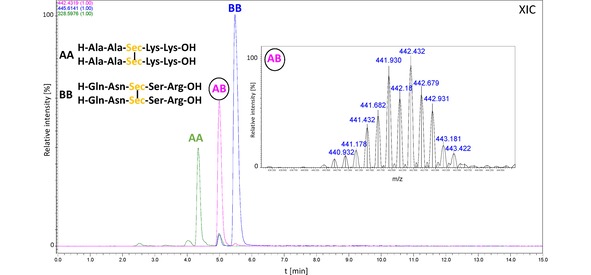
LC‐MS chromatogram (XIC) representing the mixture of peptides formed as a results of metathesis of two model diselenides (peptides marked as AA, BB).

**Figure 3:**
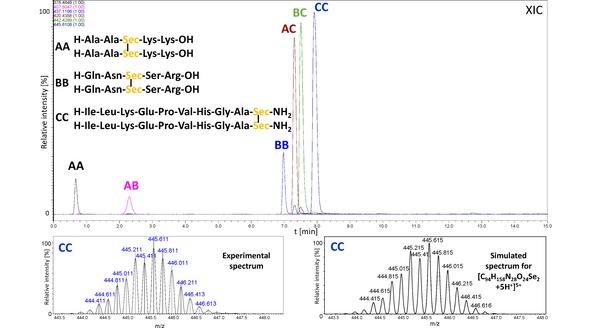
LC‐MS chromatogram (XIC) representing the mixture of peptides formed as a result of metathesis of three model diselenides (peptides marked as AA, BB, and CC). The experimental and the simulated spectra of peptide CC are presented at the bottom.

**Figure 4:**
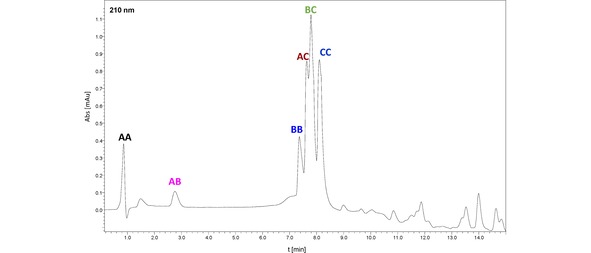
HPLC chromatogram obtained for the mixture of peptides formed as a result of metathesis of three model diselenides (peptides substrates marked as AA, BB, and CC) with detection at 210 nm.

The equimolar mixture of peptide AA and 1,2‐bis(4‐bromobenzyl)diselenide was exposed to the visible light (LED lamp) as in the previous experiments. We monitored the progress of the metathesis reaction with time and observed a gradual disappearance of the peptide substrate and formation of the target molecule. We found that prolongation of the irradiation up to 24 h resulted in a higher yield of the exchange product (Figure S12). The time progress of metathesis reaction was presented in Figure 5. Reaction observed in our system was slower, then the metathesis of 20 mM, dibenzyl diselenide ((BenSe)_2_), and 20 mM, di‐(1‐hydroxyundecyl) diselenide, which required 2 hours for equilibration21a which may be caused by lower concentration of reagents in our experiment (1 mM od each component). However, direct comparison of these results is risky, because of significant differences is structures of substrates. We confirmed also the previous literature reports that application of similar conditions enables the exchange reaction between two low‐molecular‐weight compounds.^21^ The reactions were monitored by NMR and the final results are presented in the Supplementary section (see Figures S13–14). The metathesis reaction for low‐molecular‐weight systems was also confirmed by TLC (data not presented).

**Figure 5:**
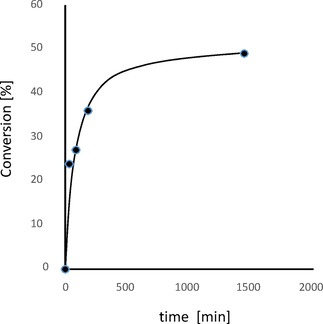
Kinetics of exchange in an equimolar mixture of peptide AA and 1,2‐bis(4‐bromobenzyl)diselenide exposed to the visible light. The graph shows percent of conversion of peptide AA as a function of time.

The exchange processes did not proceed in the absence of light. This observation is consistent with the literature data and, in our opinion, decisively proves the proposed free radical mechanism.21a Thus, the aforementioned mechanism (ref. [21a]) can be only a reasonable explanation of this process. The literature data demonstrate that Sec can be used in proteins and peptides in order to enforce the correct folding.^8^, ^9^, ^22^ Due to a lower oxidizing potential of Sec relative to Cys, the replacement of the latter by Sec prevents, at some conditions, the disulfide scrambling and, thereby, leads to a properly folded protein. Thus, data presented in this paper seems to find interest in this field, especially the study on the influence of the intramolecular metathesis on the protein folding. In conclusion, we demonstrated a facile method of light‐driven metathesis of peptides containing a diselenide bridge. We showed for the first time the visible light induced diselenides exchange in peptides. Our study can contribute to the field of protein folding or synthesis of peptide libraries based on diselenide bridges. The reaction proceeds in a visible light under mild conditions (ambient temperature). The previously reported diselenide and disulfide exchange ractions in polypeptides were based on nucleophilic mechanism, and required addition of reducing agent and in some cases the appropriate catalyst (e. g. glutathione or its selenoanalogue). The main advantage of the method described herein is the possibility of controlling (switching on/off) reactions without chemical modification of the reacting system. Further studies aimed at solving the problem of diselenide exchange in more complex systems with two or more intramolecular diselenide bonds are ongoing.

## Experimental Section

### Peptide Preparation

The model peptides containing the selenocysteine residue were prepared on a solid support according to the standard Fmoc protocol. For this purpose, we used a polypropylene syringe reactor equipped with a polyethylene filter (Intavis). The coupling of respective amino acid residues was carried out using PyBOP in DMF over 15 h. The mixture was sonicated.^23^ The Fmoc−Sec(Mob)−OH derivative was chosen to introduce the selenocysteine residue. The crude product was cleaved from the resin using the mixture of TFA/H2O/TIS (95 : 2.5 : 2.5, v/v) for 2 h at room temperature. The peptides obtained after the cleavage contained the Mob (4‐metoxybenzyl) protecting group. To remove this protection, the mixture of 5 % DMSO/TFA for 5 h at ambient temperature was used. This resulted in obtaining of an oxidized peptide containing the Se−Se bridge. The crude peptides were precipitated in cold diethyl ether and subsequently lyophilized.

### Diselenide Exchange

Oxidized peptides, containing the selenocysteine residue, were mixed at the equimolar ratio (glass vial, 1.5 ml) and dissolved in methanol (concentration of each peptide was 1 mM). Then, the sample was subjected to irradiation for at least 30 min using a LED lamp (the vial was attached directly to the light source). Finally, 50 μl of methanolic solution was taken, diluted with 450 μl of water and analyzed by LC‐MS. The experiments on diselenide exchange in peptides were also performed in acetonitrile, with very similar results (data not presented).

## Conflict of interest

The authors declare no conflict of interest.

## Supporting information

As a service to our authors and readers, this journal provides supporting information supplied by the authors. Such materials are peer reviewed and may be re‐organized for online delivery, but are not copy‐edited or typeset. Technical support issues arising from supporting information (other than missing files) should be addressed to the authors.

Supplementary
