# Supplementary material for: Light‐Driven Diselenide Metathesis in Peptides
Source: ChemistryOpen. 2019 Sep 10;8(9):1199–203. doi: 10.1002/open.201900224 (PMC6735248; doi:10.1002/open.201900224)
Supplement: Supplementary file 1 — Supplementary [file OPEN-8-1199-s001.pdf]

ChemistryOPEN
Open Access

Supporting Information

© Copyright Wiley-VCH Verlag GmbH & Co. KGaA, 69451 Weinheim, 2019

Light-Driven Diselenide Metathesis in Peptides

Mateusz Waliczek, Özge Pehlivan, and Piotr Stefanowicz*©201x The Authors. Published by Wiley-VCH Verlag GmbH & Co. KGaA.
This is an open access article under the terms of the Creative Commons Attribution License, which permits use, distribution and reproduction in any medium, provided the original work is properly cited.

A LIGTH-DRIVEN DISELENIDE METATHESIS IN PEPTIDES

Mateusz Waliczek[a], Özge Pehlivan[a], Piotr Stefanowicz[a] *

[a] *Faculty of Chemistry, University of Wrocław, F. Joliot-Curie 14, 50-383 Wroclaw, Poland*

EXPERIMENTAL

Reagents

Solvents for peptide synthesis (analytical grade) were obtained from Sigma-Aldrich (dimethylformamide-DMF; trifluoroacetic acid-TFA) and J. T. Baker (diethyl ether). The H-Rink amide Chemmatrix® resin was purchased from Sigma-Aldrich. The (benzotriazol-1-yloxy)tripyrrolidinophosphonium hexafluorophosphate (PyBOP) coupling reagent were obtained from Navoabiochem®. All standard amino acids derivatives were purchased from Peptydy.pl. Fmoc-Sec(pMeOBz) was purchased from Merck. Solvents for LC-MS and MS measurements: acetonitrile (MeCN), methanol (MeOH), formic acid (HCOOH) were purchased from VWR Chemicals.

Mass spectrometric analysis

The mass spectrometry measurements were carried out on Apex Ultra FT-ICR (Bruker, Germany) equipped in electrospray ion source (ESI) ion funnel. The mass spectrometer was calibrated before the run with the Tunemix mixture (Bruker Daltonics) by a quadratic method. All the measurements were performed in the positive ion mode. For the CID (collision induced dissociation) during the MS/MS experiments was optimized the collision energy (10–20 eV) for the best fragmentation (the voltage over the hexapole collision cell varied from 15 to 30 V). Argon was used as a collision gas. An acetonitrile/water/formic acid (50:50:0.1) mixture were used as the solvents for recording the mass spectra. The potential between the spray needle and the orifice was set to 4.5 kV.

LC-MS

The LC-MS analysis of obtained peptides were carried out on Shimadzu IT-TOF, which is hybrid system consisting of ion trap and time of flight mass analyzer. This instrument is also equipped in electrospray (ESI) ion source. The potential between the spray needle and the orifice was set to 4.5 kV. The LC system was operated with mobile phase, consisting of solvent A: 0.1% formic acid in H_2O and solvent B: 0.1% formic acid in MeCN. The following

separation conditions were used: 1)The gradient conditions (B %) were from 5 to 60% B within 15 min; 2) from 0 to 0% within 3 min following by increase to 60% within 12 min. The flow rate was 0.2 mL/min and the injection volume 1 μL. The separation was performed on a Aeris Peptide XB-C18 column (50 mm × 2.1 mm) 3.6 μm bead diameter. The samples of peptide were dissolved in 400 μl of water : acetonitrile mixture (95 : 5).

NMR analysis

The NMR measurements for low molecular weight diselenide were carried out on Bruker Avance 500 MHz spectrometer at 25 °C, at the analyte concentration of 5 mg mL^{-1}, in 99% $CDCl_3$.

*Purification and characterization of peptides***.**

The model Sec-contained peptides were purified by preparative reversed-phase HPLC on a Vydac C18 column (22 mm x 9 250 mm), using the following solvent systems: S1 0.1% aqueous TFA, S2 80% acetonitrile + 0.1% TFA, linear gradient from 5 to 70% of S2 for 50 min, flow rate 7.0 ml/min, UV detection at 210 and 280 nm. The resulting fractions were collected and subjected to a lyophilization process. The identities of the products were confirmed by MS analysis using a Apex Ultra FT-ICR (Bruker, Germany) mass spectrometer equipped with an electrospray (ESI) ionization source. The purity of peptides were analyzed using a Thermo Separation HPLC system with a UV detection (210 nm) on a Vydac Protein RP C18 column (4.6 × 250 mm, 5 μm), with a gradient elution of 0%–80% S2 in S1 (S1 = 0.1% aqueous TFA; S2 = 80% acetonitrile + 0.1%) for 40 min (flow rate 1 mL/min). The metathesis products were analyzed by HPLC on a Aeris Peptide XB-C18 column (50 mm × 2.1 mm) 3.6 μm bead diameter using Shimadzu IT-TOF instrument equipped in PDA detector. The separation conditions were described in section LC-MS.

Synthesis of 1,2-bis(4-bromobenzyl)diselane

Sodium Diselenide and 1,2-bis(4-bromobenzyl)diselane were synthesized as stated in procedure published by Klayman*et al*.[1]

<u>Sodium Diselenide:</u>

---

[1]Klayman, L. D.; Griffin, T. S. *J. Amer. Chem. Soc.*, 1973, ***95***, 197-199.

Absolute ethanol (75 mL) was added to selenium powder (1.5 g, 19 mmol) and sodium borohydride (0.5 g, 13 mmol) and allowed the mixture to cool down in ice bath for 15 minutes. After exothermic reaction, mixture was stirred and heated under reflux by purging nitrogen for 1.5 hours. Red/brown colored solution was ready to be used in the next step.

$$2NaBH_4 + 3Se + 6C_2H_5OH \longrightarrow Na_2Se_2 + H_2Se + 2B(OC_2H_5)_3 + 6H_2$$

1,2-bis(4-bromobenzyl)diselenide:

4-bromobenzyl bromide (3.1 g, 12.5 mmol) was added into ethanolic solution of sodium diselenide described above. Obtained solution was purged with nitrogen and heated under reflux for 4 hours. Green/yellow colored solution was then cooled down to room temperature. After cooling, solution was diluted with distilled water (50 mL) and extracted with chloroform (2 x 75 mL). Organic phase was dried over anhydrous magnesium sulfate, filtered and solvent was removed by rotary evaporator. Greenish-yellow product was dried further in vacuum resulting 2.5 g of mass and 80.6 % of yield. R_F= 0.78 (Hexane : EtOAc = 85 : 15), M. P. = 104.1°C -104.4 °C.

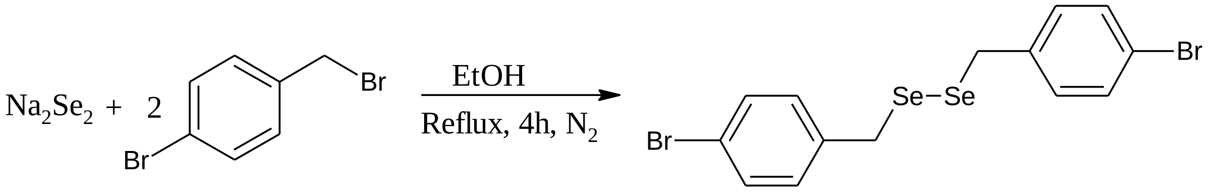

* ^{1}H NMR (500 MHz, DMSO-d$_6$); δ (ppm): 7.52-7.49 (m, 4H), 7.20-7.17 (m, 4H), 3.95–3.92 (m, 4H) (Fig. S9)
* m/z $[M+Ag]^+$ (calculated): 606.666, m/z $[M+Ag]^+$ (found): 606.664 (Fig. S10)
* $(CD_3)_2SO$ and HOD peaks are observed at 2.50 ppm and 3.31 ppm, respectively.[2]

1,2-bis(4-cyanobenzyl)diselenide:

* Synthesis of 1,2-bis(4-cyanobenzyl)diselenide was performed based on previous procedure.

Sodium diselenide was first synthesized by the reaction of selenium powder (0.6 g, 7.6 mmol) and sodium borohydride (0.2 g, 5.3 mmol) in presence of absolute ethanol (30 mL), after

[2]Gottlieb, H. E.;Kotlyar, V.; Nudelman, A. *J. Org. Chem.*, 1997, **62,** 7512-7515.

cooling in ice bath for 15 minutes. Reaction was performed by refluxing the mixture for 2 hours with nitrogen purging and yielded reddish-brown colored solution.

$$2NaBH_4 + 3Se + 6C_2H_5OH \longrightarrow Na_2Se_2 + H_2Se + 2B(OC_2H_5)_3 + 6H_2$$

After addition of 4-bromomethyl benzonitrile (0.98 g, 5 mmol) into sodium diselenide solution, it was heated under reflux for 5 hours in presence of nitrogen gas and then cooled down to room temperature. Solution was washed with distilled water (20 mL) and chloroform (2 x 30 mL). Organic phase was dried over anhydrous magnesium sulfate, filtered and solvent was removed by rotary evaporator. Orange colored product was dried in vacuum and resulted 62 % of yield with 0.61 g of mass. R_F = 0.14 (Hexane : EtOAc = 85 : 15), M. P. =136.7°C - 138.3°C.

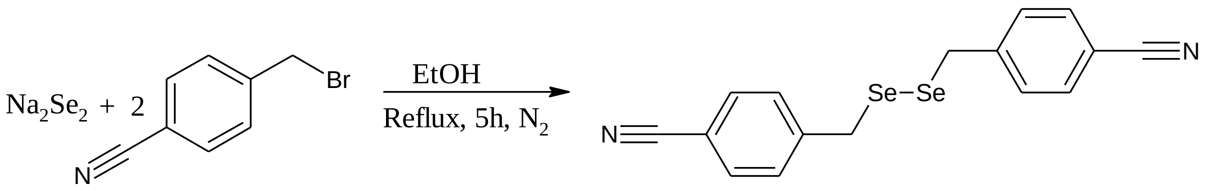

^{1}H NMR (500 MHz, DMSO-d$_6$); δ (ppm): 7.79-7.76 (m, 4H), 7.42-7.39 (m, 4H), 4.05–4.02 (m, 4H) (Fig. S13)

* $(CD_3)_2SO$ and HOD peaks are observed at 2.50 ppm and 3.31 ppm, respectively.[2]

Diselenide Exchange Reaction:

1,2-bis(4-bromobenzyl)diselane (9.96 mg, 0.02 mmol) and 1,2-bis(4-cyanobenzyl)diselenide (7.80 mg, 0.02 mmol) were mixed in $CDCl_3$(1 mL). Orange colored solution was then irradiated with visible light for 1 hour. R_F(upper)= 0.77, R_F (middle) = 0.43, R_F (lower) = 0.16.

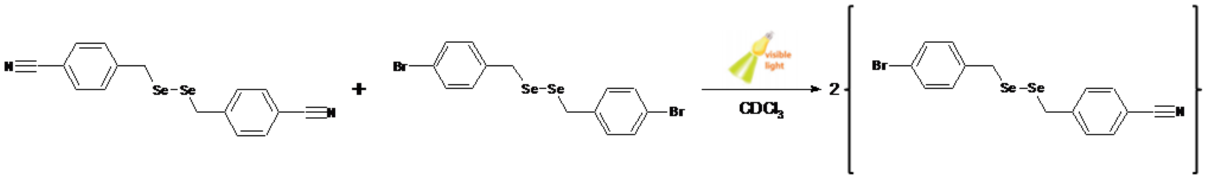

MS, NMR spectra and chromatograms

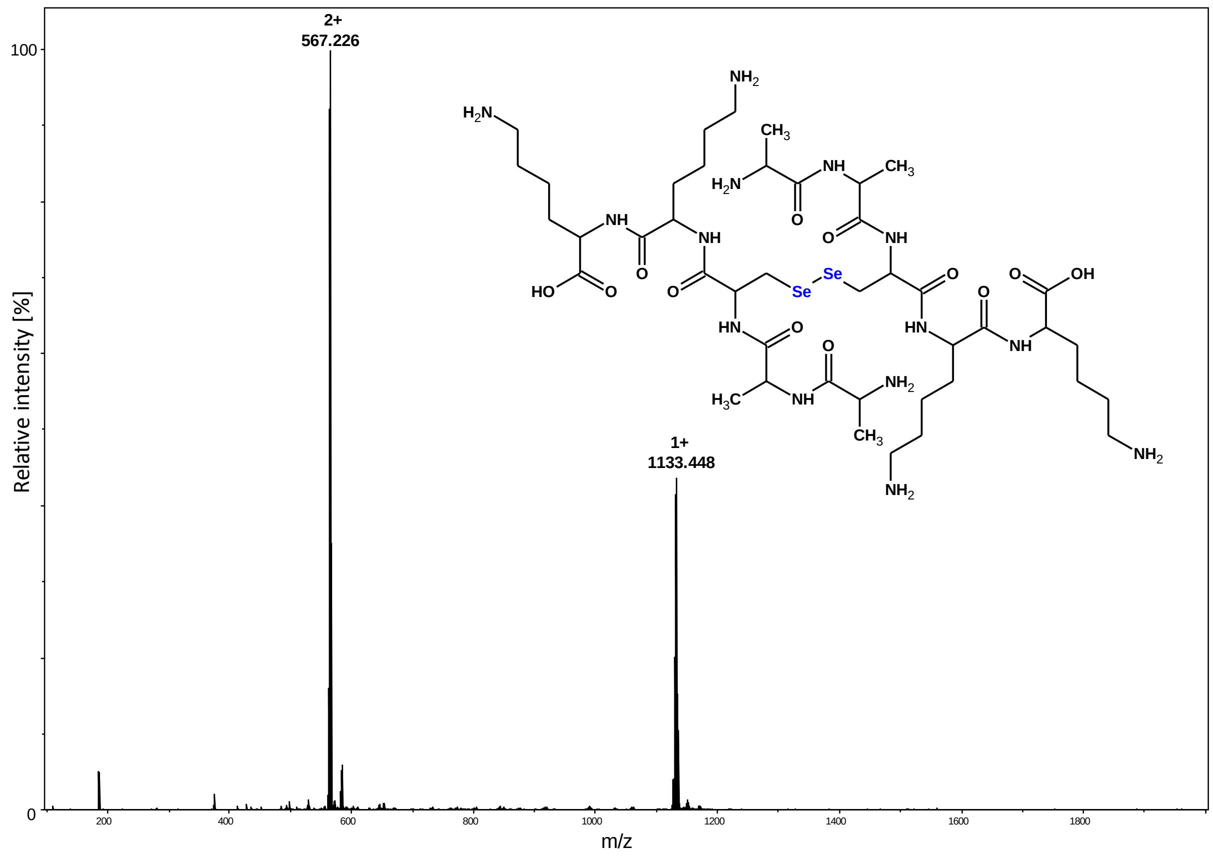

Fig S 1. ESI-MS spectrum obtained for the purified peptide (H-Ala-Ala-Sec-Lys-Lys-OH)$_2$

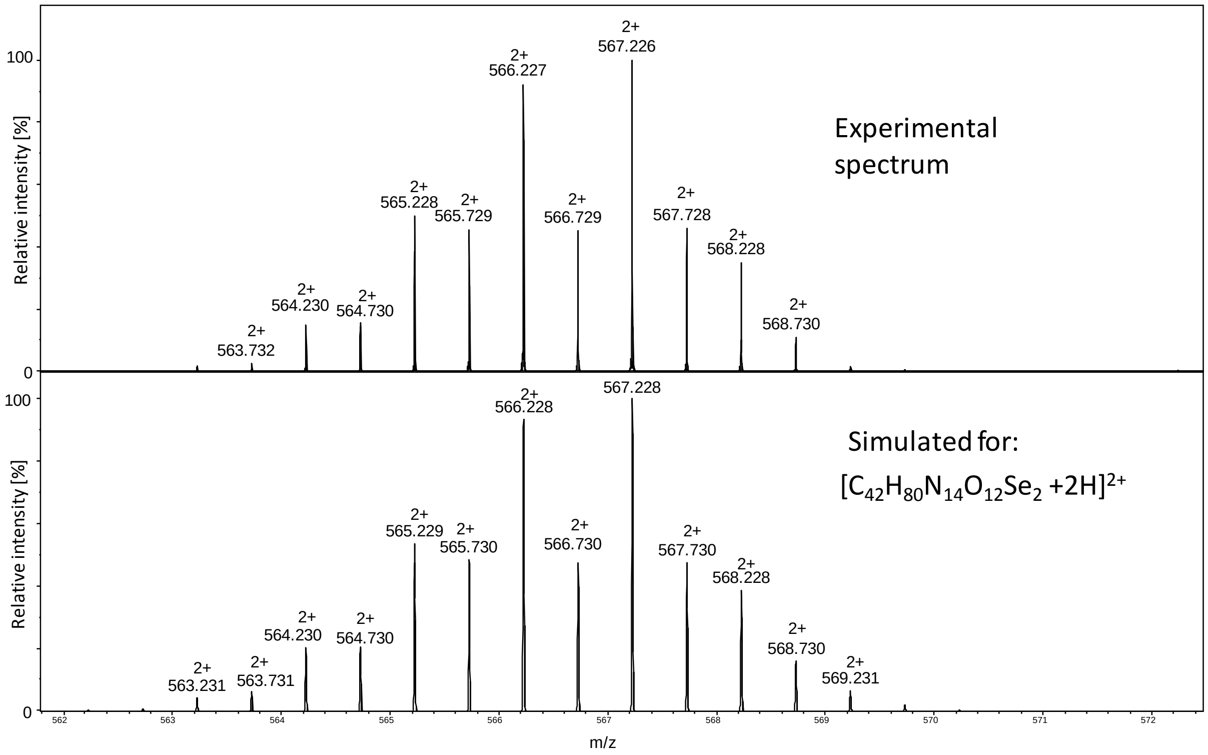

Fig S 2. ESI-MS spectrum obtained for the purified peptide (H-Ala-Ala-Sec-Lys-Lys-OH)$_2$ – expanded area of signal $[M+2H]^{2+}$ with characteristic isotopic pattern.

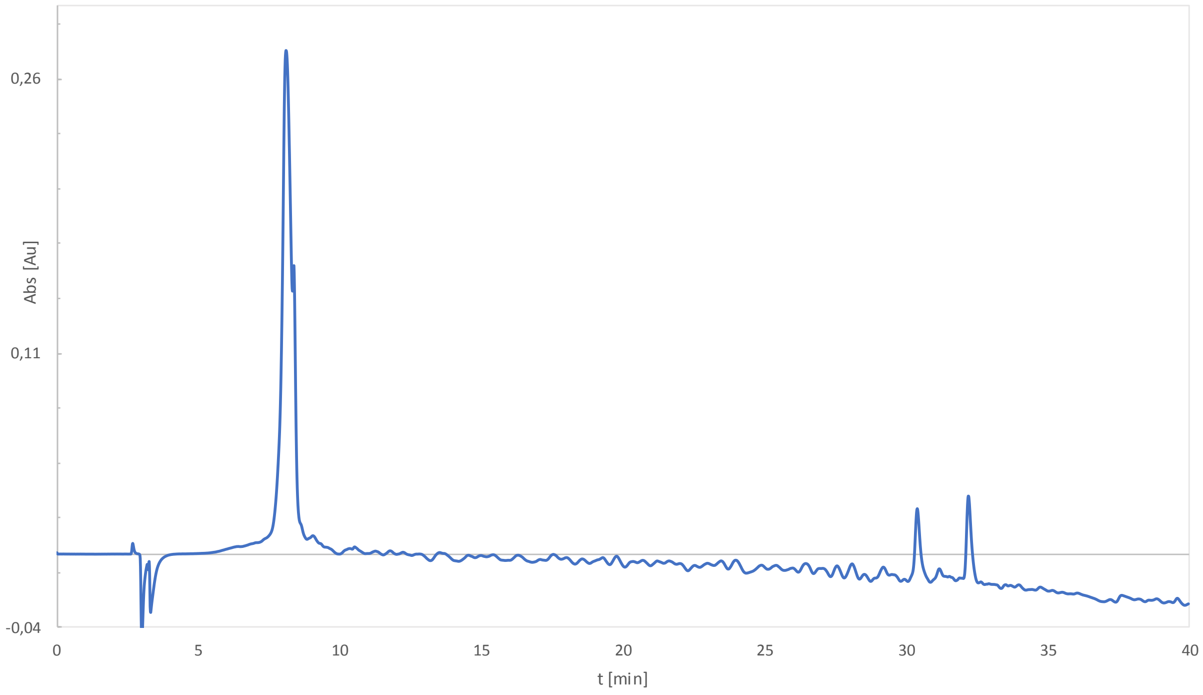

Fig S 3. HPLC chromatogram obtained for peptide (H-Ala-Ala-Sec-Lys-Lys-OH)$_2$

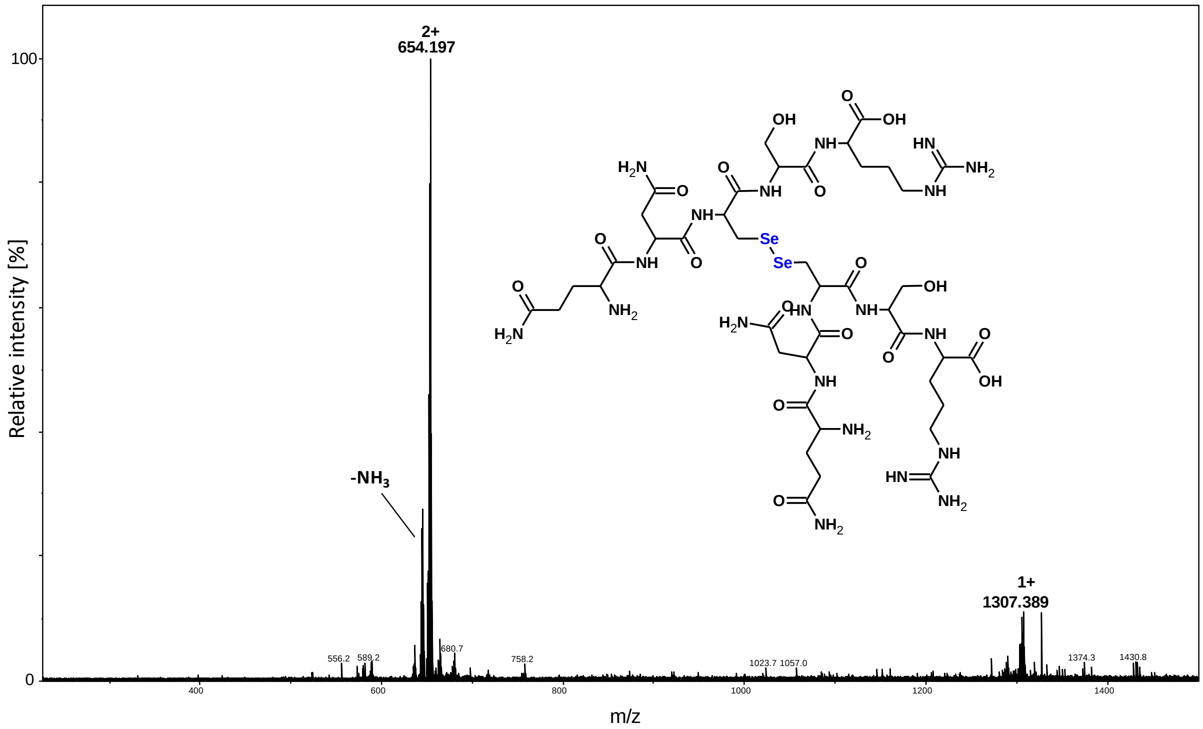

Fig S 4. ESI-MS spectrum obtained for the purified peptide (H-Gln-Asn-Sec-Ser-Arg-OH)$_2$

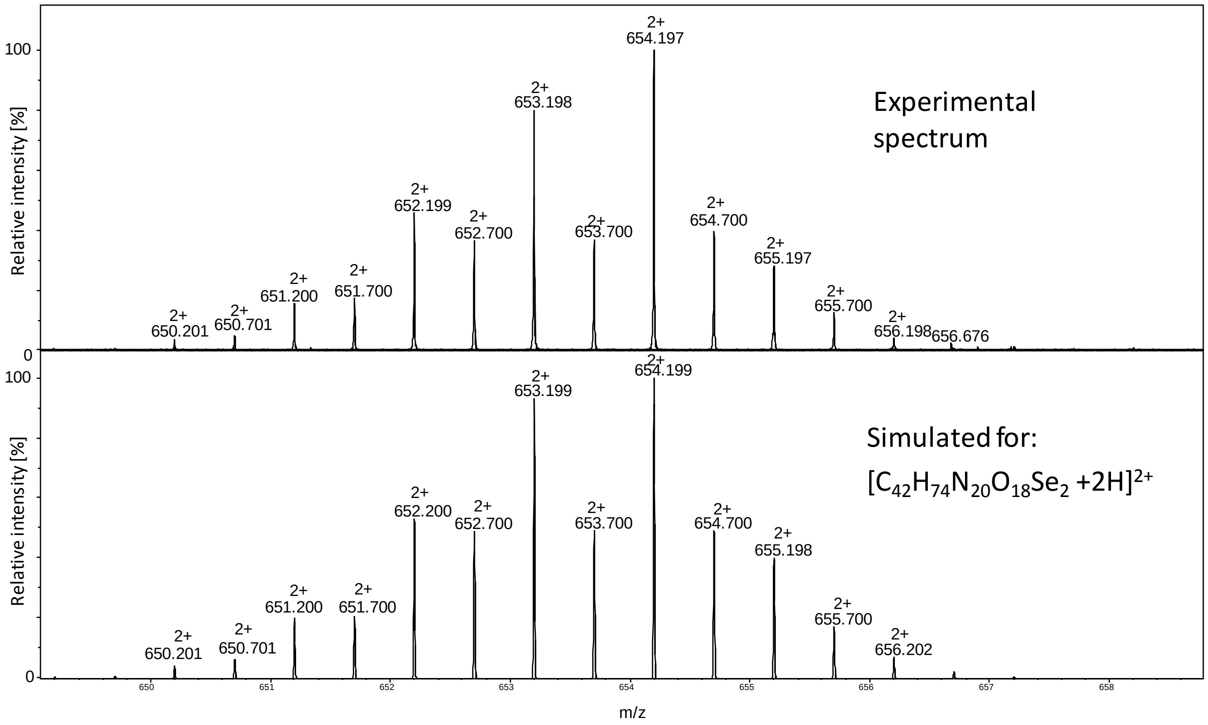

Fig S 5. ESI-MS spectrum obtained for the purified peptide (H-Gln-Asn-Sec-Ser-Arg-OH)$_2$ – expanded area of signal $[M+2H]^{2+}$ with characteristic isotopic pattern.

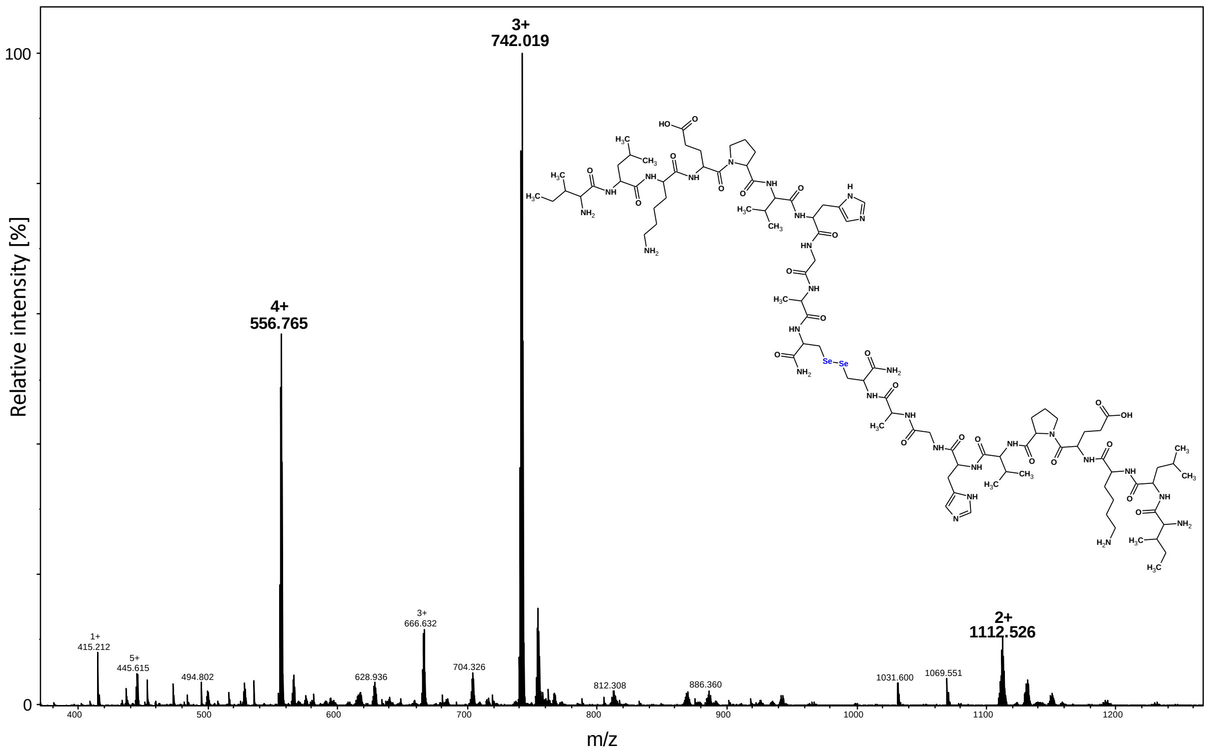

Fig S 6. ESI-MS spectrum obtained for the purified peptide (H-Ile-Leu-Lys-Glu-Pro-Val-His-Gly-Ala-Sec-NH$_2$)$_2$

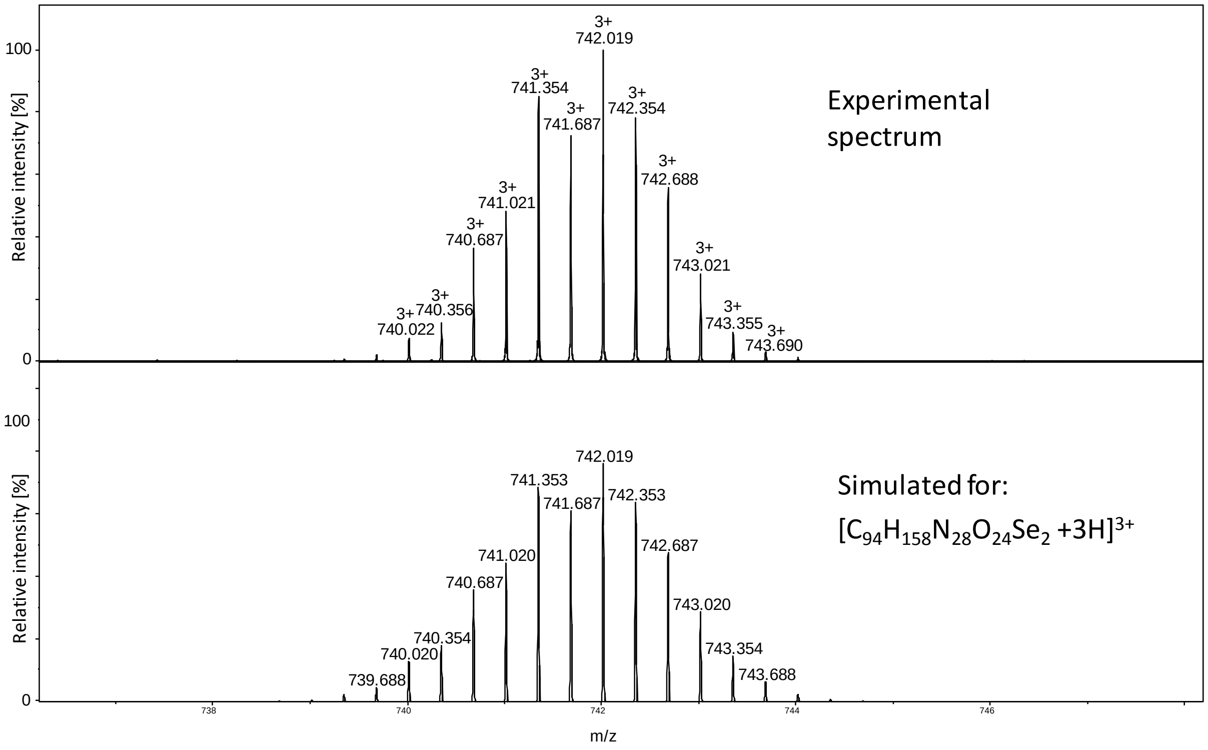

Fig S 7. ESI-MS spectrum obtained for the purified peptide (H-Ile-Leu-Lys-Glu-Pro-Val-His-Gly-Ala-Sec-NH_2)$_2$ – expanded area of signal $[M+3H]^{3+}$ with characteristic isotopic pattern.

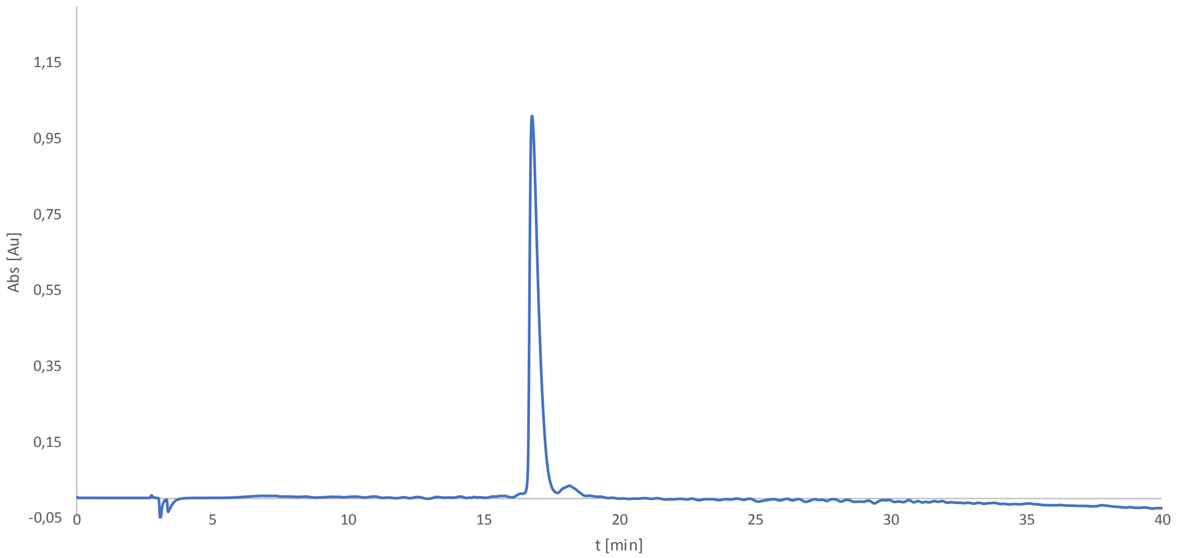

Fig S 8. HPLC chromatogram obtained for the purified peptide (H-Ile-Leu-Lys-Glu-Pro-Val-His-Gly-Ala-Sec-NH_2)$_2$

.

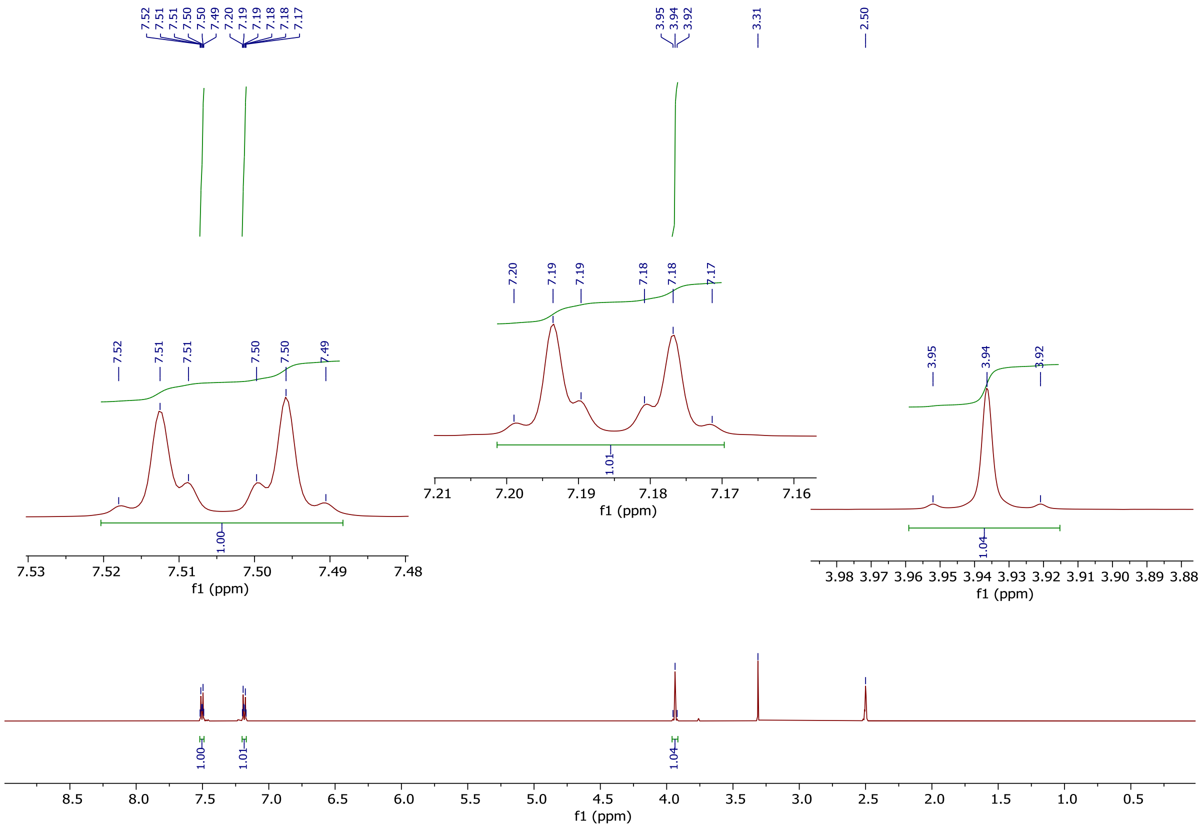

Fig S 9. ^{1}H NMR spectrum of 1,2-bis(4-bromobenzyl)diselenide in DMSO-d_6

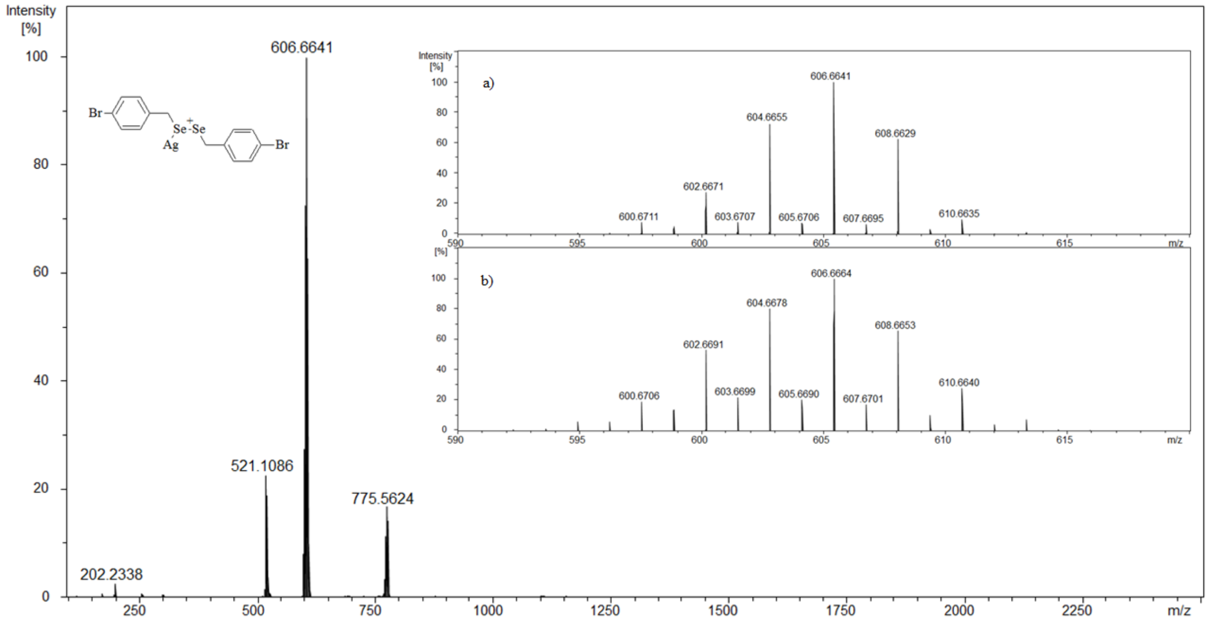

Fig S 10. Mass spectrum of 1,2-bis(4-bromobenzyl)diselanide (in methanol with addition $AgNO_3$). a) Isotopic distribution of relative peak corresponding to 1,2-bis(4-bromobenzyl)diselenide b) Isotopic distribution of simulated peak corresponding to 1,2-bis(4-bromobenzyl)diselenide.

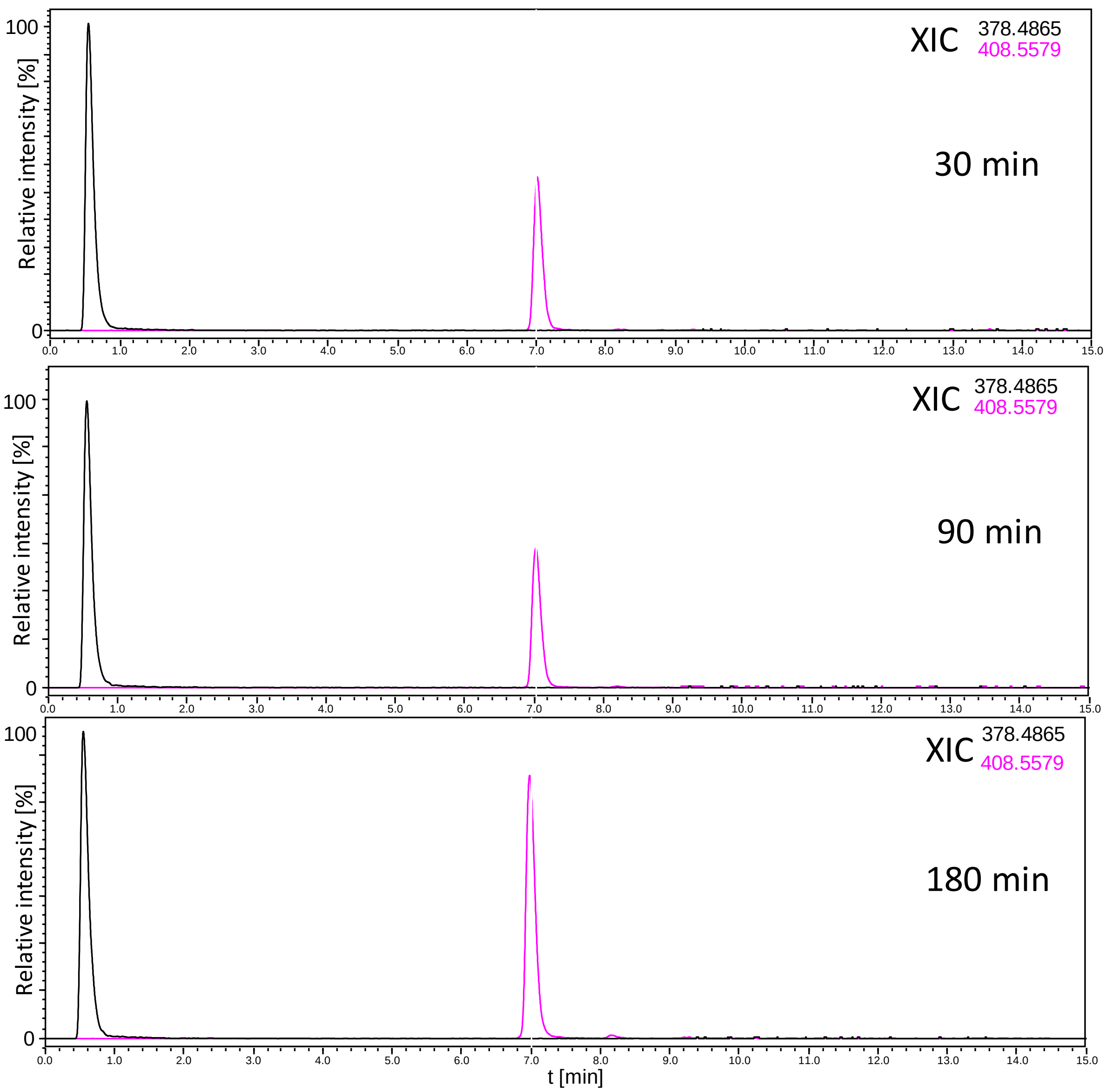

Fig S 11. LC-MS chromatograms (XIC) showing the progress of reaction between the peptide H-Ala-(Ala-Sec-Lys-Lys-OH)$_2$ and 1,2-bis(4-bromobenzyl)diselane. The fast eluting signal derived from the pure peptide, while the second one represents the hybride of peptide and the low molecular weight compounds.

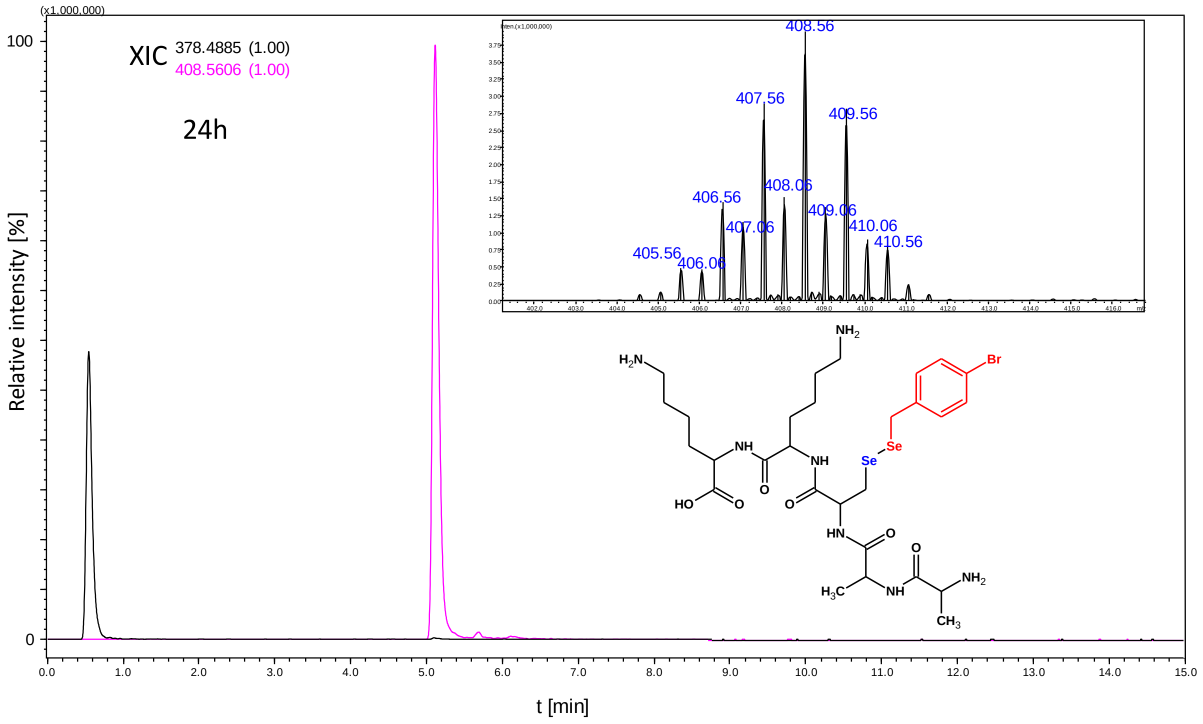

Fig S 12. LC-MS chromatogram (XIC) obtained for the product of exchange reaction between the peptide (H-Ala-Ala-Sec-Lys-Lys-OH)$_2$ and 1,2-bis(4-bromobenzyl)diselenide after 24h. The fast eluting signal derived from the pure peptide, while the second one represents the hybride of peptide with low molecular weight compound. In the figure was also shown the expanded area of MS signal obtained for the product.

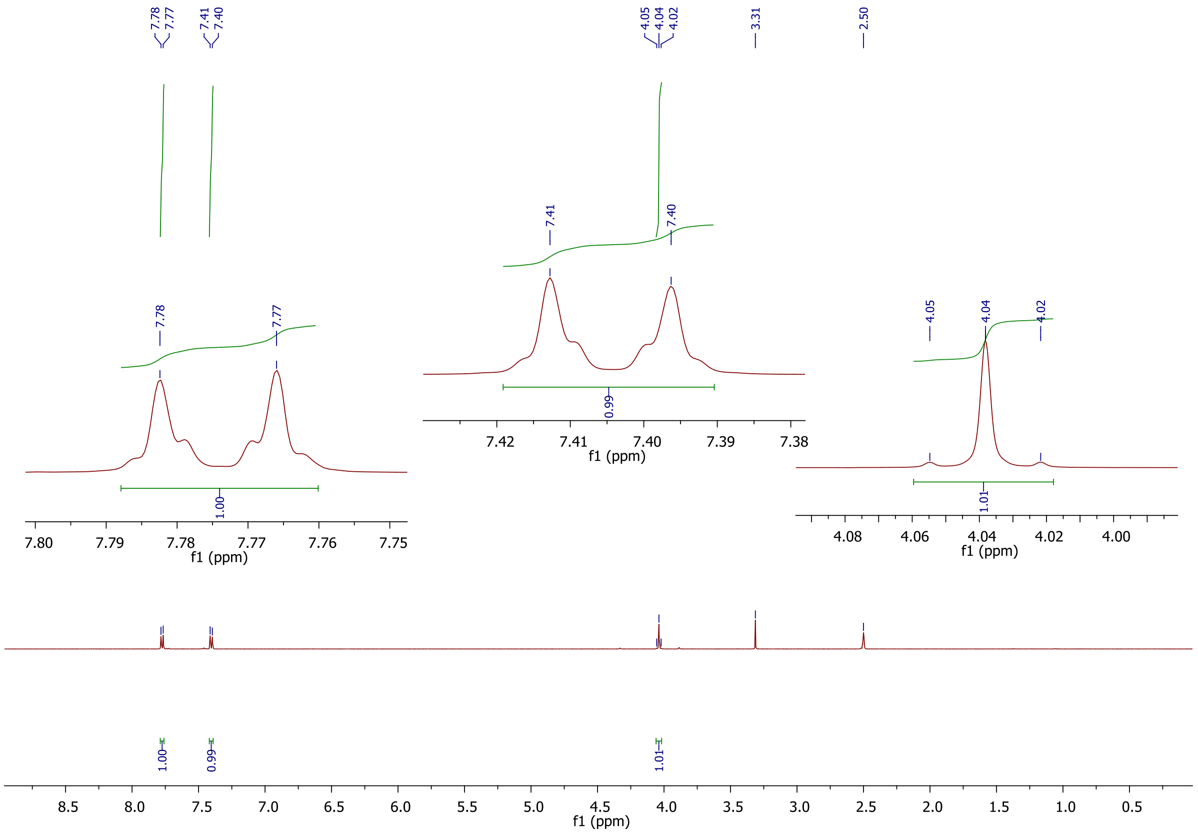

Fig S 13. 1H NMR spectrum of 1,2-bis(4-cyanobenzyl)diselenide in DMSO-d$_6$

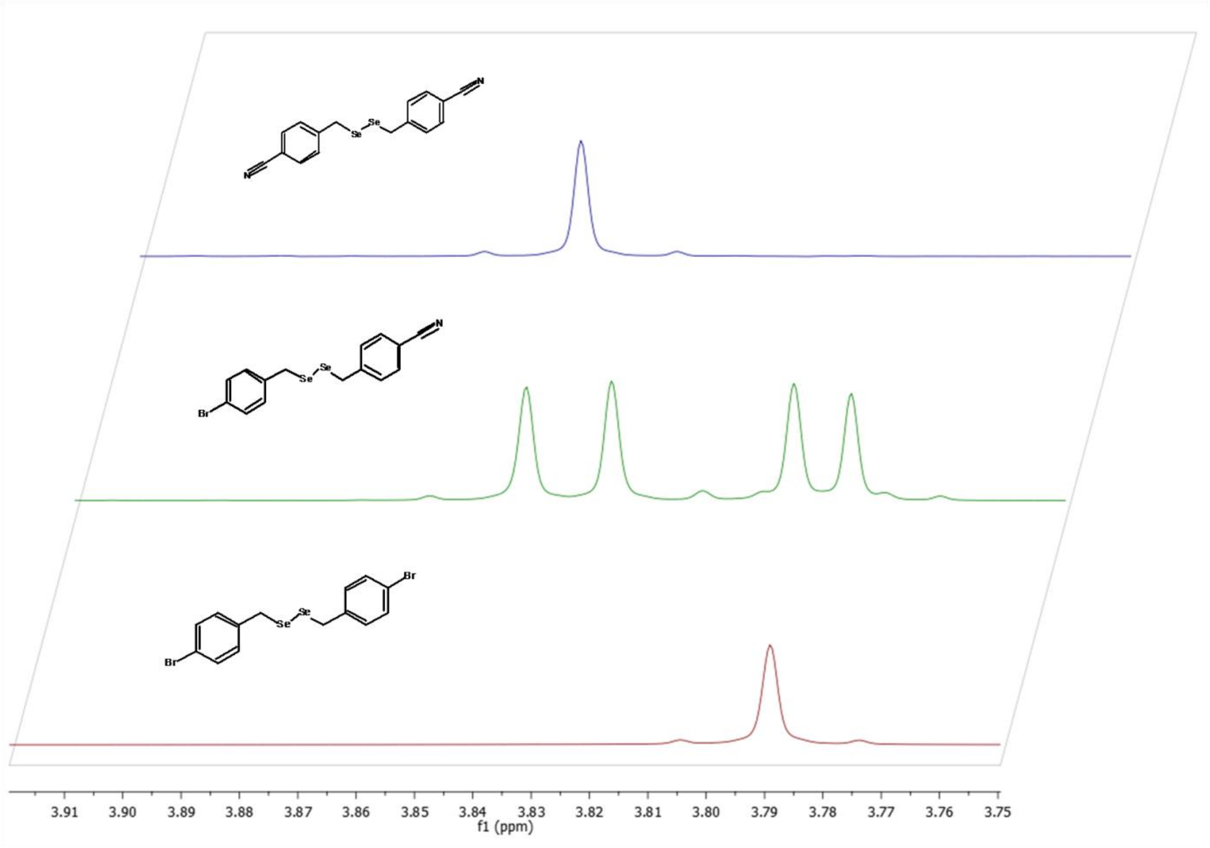

Fig. S 14 ^{1}H NMR spectrum- stacked representation of 1,2-bis(4-bromobenzyl)diselenide (purple), exposed to light mixture of 1,2-bis(4-bromobenzyl)diselenide, and 1,2-bis(4-cyanobenzyl)diselenide (green) and 1,2-bis(4-cyanobenzyl)diselenide (blue) in $CDCl_3$

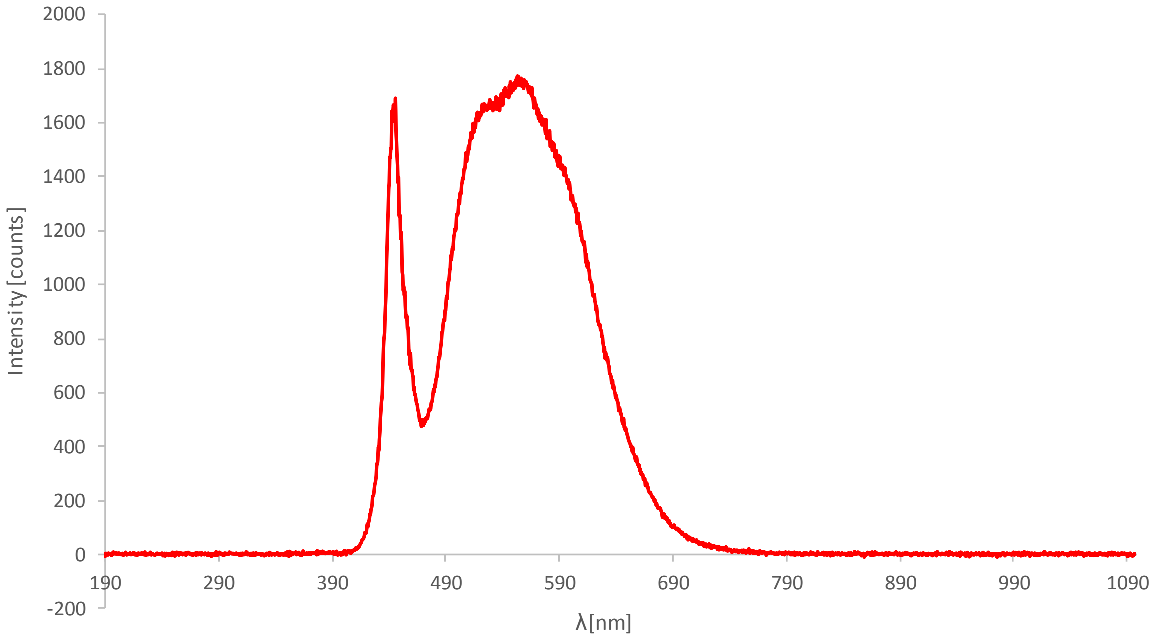

Fig. S 15. Emission spectrum measured for LED lamp.
